# Benchmark Response Values for Error‐Corrected Sequencing Mutagenicity Assessment Technologies

**DOI:** 10.1002/em.70051

**Published:** 2026-04-27

**Authors:** Saron Mulugeta, Francesco Marchetti, Guangchao Chen, Connie Chen, Raechel Puglisi, Paul A. White

**Affiliations:** ^1^ School of Computer Science, Carleton University Ottawa Ontario Canada; ^2^ Environmental Health Science and Research Bureau, Environmental and Radiation Health Sciences Directorate, Health Canada Ottawa Ontario Canada; ^3^ Department of Biology Carleton University Ottawa Ontario Canada; ^4^ Centre for Prevention, Lifestyle and Health, RIVM (National Institute for Public Health and the Environment) Bilthoven the Netherlands; ^5^ Health and Environmental Sciences Institute (HESI) Washington, DC USA

**Keywords:** benchmark dose, duplex sequencing, effect size, HAWK‐seq, PacBio HiFi, PECC‐seq, PROAST, risk assessment

## Abstract

The Benchmark Dose (BMD) approach is commonly used to determine Point‐of‐Departure (PoD) values for risk assessment and regulatory decision‐making; however, choosing a suitable Benchmark Response (BMR) for continuous endpoints is a challenge. Earlier work established a BMR of 50% for selected in vivo mutagenicity endpoints (i.e., Transgenic Rodent and *Pig‐a*). Error‐corrected sequencing (ECS) technologies, such as Duplex Sequencing (DupSeq), Hawk‐Seq, PECC‐Seq, and PacBio HiFi, have emerged as powerful tools for mutagenicity assessment. This study applied and compared two approaches for defining BMR values for ECS technologies: the Effect Size (ES) theory of Slob (2017), and the one standard deviation approach of Zeller et al. (2017). A dose–response database of ECS studies was compiled to determine technology‐specific within‐group variance values (*var*) for BMR determination. Experimental factor influences on *var*, including species, rodent strain, administration route, application time, tissue type, tissue sampling time, and DNA fragmentation method, were examined; no significant influences were detected. The absence of covariate effects justified using typical, technology‐specific *var* values for BMR determinations. Using these values, technology‐specific BMRs were calculated as 27.7% for DupSeq, 16.6% for Hawk‐Seq, and 23.3% for PECC‐Seq. BMRs derived from negative control values were 22.6 to 28.8% for DupSeq, 5.6 to 13.8% for Hawk‐Seq, 28.7 to 31.5% for PECC‐Seq, and 9.5 to 22.8% for PacBio HiFi. These findings support adoption of a 30% BMR for in vivo ECS mutagenicity assessment technologies, providing a robust and consistent foundation for future dose–response modeling and human health risk assessment.

## 1 Introduction

Error‐corrected next‐generation sequencing (ECS) technologies, such as Duplex Sequencing (DupSeq), Hawk‐Seq, PECC‐Seq, and PacBio HiFi sequencing, permit highly sensitive detection of rare mutations (You et al. 2020; Marchetti et al. 2023; Matsumura et al. 2025; Miranda and Revollo 2024). They are increasingly being used for assessment of mutagenic hazard (Marchetti et al. 2023; Menon and Brash 2023); indeed, their ability to quantify mutations across diverse chemicals, species, tissues and exposure conditions makes them powerful tools for applied genotoxicity assessment (Yauk et al. 2026). However, to integrate these technologies into quantitative risk assessment and regulatory decision‐making, there is a requirement to scrutinize their utility for determination of mutagenic potency.

The Benchmark Dose (BMD) approach is widely recognized as a robust and flexible method for defining points of departure (PoDs) in toxicology (Crump 1984; Haber et al. 2018; Slob 2002; White et al. 2020; Slob et al. 2025). BMD values can support margin of exposure (MOE) calculations and, through extrapolation, determination of human exposure limits such as tolerable daily intakes (TDIs) and permitted daily exposures (PDEs) (Johnson et al. 2014; Chepelev et al. 2023; White et al. 2020; Simon et al. 2025). Central to this framework is the Benchmark Response (BMR), which specifies the magnitude of change in response considered biologically adverse. For continuous endpoints, this is typically expressed as a percent change from control (background) levels. Because the chosen BMR directly influences the resulting BMD, its selection is critical for regulatory interpretation of toxicological dose–response data (Haber et al. 2018; USEPA 2012; EFSA Scientific Committee 2022).

Different strategies for BMR determination (for continuous endpoints) have been proposed. The US Environmental Protection Agency (US EPA) provides several options for BMR specification for continuous data; defining the BMR as one standard deviation (1SD) above the negative control mean is generally included in the analysis (USEPA 2012, 2022). However, the 1SD approach yields values that are implicitly dependent on background variability (Haber et al. 2018; White et al. 2020). The European Food Safety Authority (EFSA) initially recommended a default BMR of 5% for continuous endpoints (Hardy et al. 2017), later shifting to a tiered approach that incorporates biological considerations or statistical principles such as the Slob (2017) effect size (ES) theory (EFSA Scientific Committee 2022). The ES theory establishes an empirical link between within‐group variance (*var*) and the maximum fold‐change in response, enabling endpoint‐specific BMRs to be derived directly from *var* estimates, that is, surrogates of the maximum fold‐change. Complementary work by Zeller et al. (2017) used large‐scale analyses of historical control values to argue that realistic BMRs for genotoxicity endpoints should quantitatively reflect endpoint variability.

Despite these frameworks, no formal consensus exists for genotoxicity endpoints; the literature shows considerable variability with BMR values ranging from 10% to 79% depending on the endpoint and context (Zeller et al. 2017; White et al. 2020; Johnson et al. 2021; Chepelev et al. 2023). In practice, a BMR of 50% has often been applied for in vivo genotoxicity endpoints (e.g., Gollapudi et al. 2020; Johnson et al. 2021; Marchetti et al. 2021; Douglas et al. 2022; Bercu et al. 2023; Chepelev et al. 2023; Lynch et al. 2024; Powley et al. 2024; Zhang et al. 2024, 2025; Roper et al. 2025; and Simon et al. 2025). Importantly, 50% was recently recommended by White et al. (2025) for the *Pig‐a* and Transgenic Rodent (TGR) in vivo mutagenicity endpoints. This value has recently been extended to ECS mutagenicity studies (Bercu et al. 2023; Dodge et al. 2023; Zhang et al. 2024, 2025; Schuster et al. 2024; Lynch et al. 2024; LeBlanc et al. 2025; Simon et al. 2025; Roper et al. 2025). However, since no technology‐specific analysis has hitherto been conducted, it is not clear whether this BMR is appropriate for ECS technologies.

The present study, which was conducted under the auspices of the Genetic Toxicology Technical Committee (GTTC) of the Health and Environmental Sciences Institute (HESI), addresses this gap by applying the two aforementioned approaches (i.e., the ES theory and the 1SD approach) to a curated ECS dose–response database. Mutagenicity dose–response data were compiled for ECS technologies with available in vivo mutagenicity dose–response data (i.e., DupSeq, Hawk‐Seq, PECC‐Seq, and PacBio HiFi sequencing) and analyzed using the PROAST software. We first evaluated whether experimental factors such as species, strain, administration route, application time, tissue type, tissue sampling time, and DNA fragmentation method, influenced *var*. As no consistent effects were identified, typical *var* values for each technology were used to determine appropriate BMR values. BMRs were also determined using the 1SD approach; specifically, appropriate BMR values were aligned with variability in (historical) negative control values. Finally, BMR values calculated by the different approaches were employed to determine consensus recommendations for ECS technologies for in vivo assessment of mutagenic hazard. These BMR values can be used to support future dose–response analyses for human health risk assessment.

## 2 Methods

Dose–response data for selected ECS technologies (i.e., DupSeq, Hawk‐Seq, and PECC‐Seq) were obtained from both peer‐reviewed and unpublished studies, and compiled into an Excel database. To be included in the database, dose–response data for each tested chemical was required to have its own matched negative control group. The database included 47 DupSeq datasets, including 26 published datasets (LeBlanc et al. 2022, 2025; Dodge et al. 2023; Bercu et al. 2023; Smith‐Roe et al. 2023; Valentine et al. 2020; Zhang et al. 2024, 2025; Schuster et al. 2024; Stewart et al. 2025; Armijo et al. 2023; Simon et al. 2025; Xia et al. 2025; Ashford et al. 2025), and 21 unpublished datasets. With respect to the other ECS technologies, the database included 6 Hawk‐Seq datasets (Matsumura et al. 2019, 2025), and 3 PECC‐Seq datasets (You et al. 2023). PacBio HiFi data (Miranda and Revollo 2024) were excluded from the dose–response analyses because the only available study used a shared negative control group across multiple test chemicals (i.e., did not meet the dataset inclusion criteria). Nevertheless, the HiFi negative control data were used for BMR determination following the 1SD approach (see below). All data were recorded at the individual animal level, with mutation frequency (MF) values expressed as mutations per 10^8^ base‐pairs. For DupSeq datasets, the recorded values are mutation frequency minimum (MFMin), which reflect the frequency of unique (clonally corrected) mutations (Dodge et al. 2023). Strictly speaking, MF endpoints are not continuous. However, if the number of base‐pairs examined is very large (e.g., 10^8^), then MF values become very finely spaced and they can be treated as continuous for dose–response analysis.

All doses were expressed as mg/kg BW/day; where necessary, age‐specific body weight values were obtained from the scientific literature. Additionally, unit conversions required for inhalation, food, and drinking water exposures were carried out as briefly described in White et al. (2025). In instances where the animals were not treated daily, the cumulative dose delivered over the course of the study was divided by the duration of the treatment. This provided a best estimate of daily dose. The complete database is provided in Tables S1–S3.

Before determining technology‐specific BMR values using the ES approach, we assessed whether key experimental factors (covariates), including species, strain, administration route, application time, tissue type, post‐exposure tissue sampling time, and DNA fragmentation method, influenced *var*. For example, regarding the tissue sampling time, datasets were grouped by time points (e.g., 1 day, 2 days, 3 days, etc.), and a *var* estimate was derived for each group. These estimates were then compared to evaluate whether sampling time influenced *var*. This evaluation helped establish whether it was suitable to assume a single *var* across datasets within each technology.

To analyze dose–response relationships, we used PROAST Model 15 (v71.1, www.rivm.nl/en/proast), a four‐parameter exponential model:

(1) $$\;y=a\cdot c^{1-e^{-{\left(\frac{x}{b}\right)}^{d}}}$$

where *x* represents the dose, *y* is the response, *a* corresponds to the mean background response (dose = 0), *b* is the potency parameter, *c* is the maximum fold increase over background, and *d* is the log steepness of the curve (White et al. 2025; Slob et al. 2025).

Following the covariate evaluation, to generate technology‐specific estimates of *var*, PROAST Model 15 was re‐run with parameters *a* and *b* set as dataset‐dependent. Using the estimated *var* values, or more precisely the within‐group standard deviation (*s*) values (i.e., square roots of *var*), technology‐specific BMR values were calculated according to the aforementioned ES theory. Slob (2017) presents an empirical relationship between *c* and *s* across 27 toxicological endpoints, deriving a proportionality constant of 7 with a 90% confidence interval of 5.9–8.9. This proportionality constant was employed for calculation of technology‐specific BMR values according to the following equation:

(2) $$\mathrm{BMR}=e^{\left(\frac{1}{8}\cdot 7\cdot s\right)}-1$$

In parallel, the 1SD approach (USEPA 2012) was applied, where BMRs were derived from background (control) variability. Following Zeller et al. (2017), three measures of SD were considered: study‐specific control SD values, truncated historical control SD values, and the median absolute deviation (MAD) of historical control values. With respect to the truncated historical controls, the distribution of control values was trimmed by excluding the uppermost 5% to reduce the influence of outliers. These analyses were conducted using MS Excel. It should be noted that all collected control values are per‐animal values for each individual experiment of each cited study.

Data visualization was accomplished using PROAST v71.1 and the ggplot2 package (version 3.5.2) in RStudio (Posit Team 2025).

## 3 Results

To determine appropriate BMR values for the ECS technologies investigated, the analyses were conducted in two stages. First, we examined whether experimental factors influenced within‐group variance (*var*). Using technology‐specific *var* estimates, we then calculated technology‐specific BMR values according to Equation (2). In the second stage of the analyses, we examined technology‐specific background (control) values and calculated BMR values according to the approach described by Zeller et al. (2017).

Dose–response datasets were collated and curated for four ECS technologies: DupSeq (47 datasets), Hawk‐Seq (6 datasets), PECC‐Seq (3 datasets), and HiFi (4 datasets). For each technology, details about experimental factors such as species, strain, administration route, application time, tissue type, tissue sampling time, and DNA fragmentation method are summarized below in Tables 1, 2, 3, 4. With respect to the BigBlue species noted in Table 1 (i.e., mouse and rat), all studies used homozygous animal strains.

**TABLE 1:** Overview of DupSeq dose–response data used for BMR determination (47 datasets).

Experimental factor	Levels of experimental factors
Species	Mouse, rat
Strain	BigBlue mouse, BigBlue rat, MutaMouse, Tg‐rasH2 mouse, Sprague Dawley rat, NMRI mouse, *gpt* delta mouse
Tissues	Bone marrow, liver, lung, stomach, spleen, seminiferous tubules, blood, kidney
Administration route	Gavage, intraperitoneal injection (*ip*)
Application Time (days)	1, 3, 28, 70, 72, 90, 180
Tissue Sampling time (days)	1, 2, 3, 24, 28, 42, 70
DNA Fragmentation method	Sonication, enzymatic

**TABLE 2:** Overview of Hawk‐Seq dose–response data used for BMR determination (6 datasets).

Experimental factor	Levels of experimental factors
Species	Mouse
Strain	*gpt* delta mouse
Tissues	Bone marrow, liver, kidney
Administration route	Intraperitoneal injection (*ip*), oral (*po*)
Application Time (days)	5
Tissue Sampling time (days)	7
DNA Fragmentation method	Sonication

**TABLE 3:** Overview of PECC‐Seq dose–response data used for BMR determination (3 datasets).

Experimental factor	Levels of experimental factors
Species	Mouse
Strain	*gpt* delta mouse, C57BL/6J mouse, DBA2 mouse
Tissues	Liver, kidney
Administration route	Intraperitoneal injection (*ip*), oral (*po*)
Application Time (days)	4, 28
Tissue Sampling time (days)	3, 14
DNA Fragmentation method	Sonication

**TABLE 4:** Overview of HiFi dose–response data used for BMR determination (4 datasets).

Experimental factor	Levels of experimental factors
Species	Mouse
Strain	C57BL/6NHsd mouse
Tissues	Liver, kidney, lung, spleen
Administration route	Gavage
Application Time (days)	1
Tissue Sampling time (days)	56
DNA Fragmentation method	Mechanical

**FIGURE 1:**
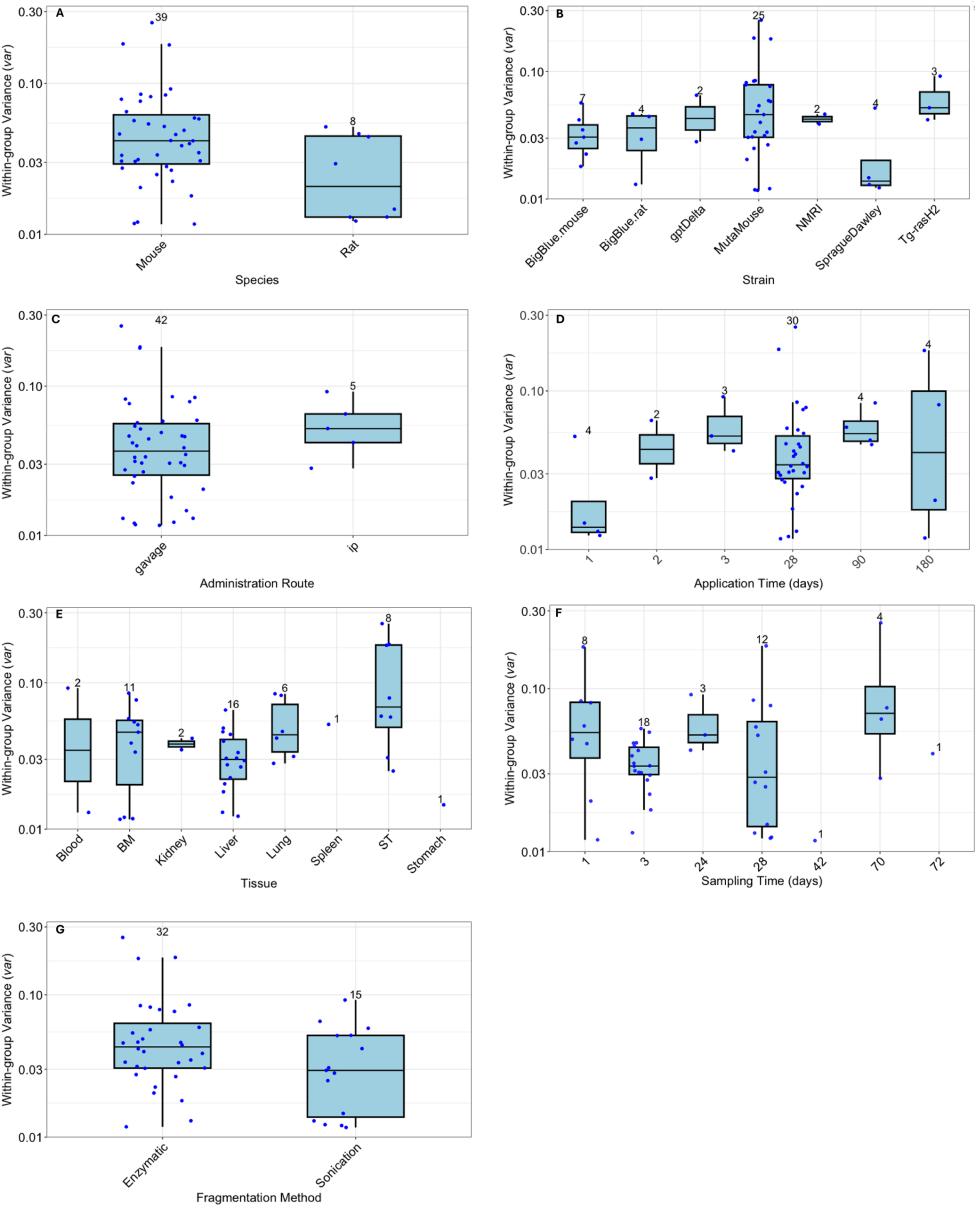
The influence of species (A), rodent strain (B), administration route (C), application time (D), tissue (E), post‐exposure tissue sampling time (F), and DNA fragmentation method (G) on within‐group variance (*var*) for the DupSeq data. The boxes and accompanying whiskers illustrate the distribution of *var* values. The upper and lower limits of the boxes indicate the interquartile range (i.e., 25th to 75th percentiles), the solid line indicates the median, and the bar extremes indicate the upper and lower 90% confidence limits. Each point represents an individual dataset, and the numbers above the bars indicate the number of datasets within each covariate category.

Before determining technology‐specific BMR values using the ES theory, we first assessed whether the experimental factors (Table 1) influenced *var*. This step was crucial for establishing if single *var* values can be assumed across datasets for each technology. Although it would be informative to conduct these analyses for all the ECS technologies examined, too few datasets are currently available for PECC‐Seq, Hawk‐Seq, and HiFi. Thus, we focused on DupSeq. Below we describe the influence of the various experimental factors on *var*; Figure 1 illustrates the distribution of the obtained *var* values across the examined factors.

The DupSeq datasets included two species: rat (8 datasets) and mouse (39) (Table 1). Mouse was the dominant species, comprising over 80% of the available datasets. Although there were fewer rat datasets, their *var* values fell within the same range as those observed for the mouse datasets, that is, value ranges overlap. Overall, there was no evidence that species influences *var* for the DupSeq datasets (Figure 1A).

The DupSeq data included seven different rodent strains: Sprague Dawley rat (SD, 4 datasets), BigBlue rat (4), BigBlue mouse (7), NMRI mouse (2), *gpt* Delta mouse (2), Tg‐rasH2 mouse (3), and MutaMouse (25) (Table 1). MutaMouse was the most frequently used rodent strain, comprising over 50% of the available datasets. Overall, there was no clear evidence that rodent strain influences *var* for the DupSeq datasets (Figure 1B).

The DupSeq datasets included two administration routes: gavage (42 datasets), intraperitoneal injection (*ip*, 5 datasets) (Table 1). Gavage was the most common route, comprising almost 90% of the available datasets. Variance estimates for *ip* were within a similar range as those observed for gavage. Overall, there was no evidence that administration route influences *var* for the DupSeq datasets (Figure 1C).

The DupSeq datasets covered a range of application times: 1 day (4 datasets), 3 days (6), 28 days (26), 70 days (2), 72 days (1), 90 days (4), and 180 days (4) (Table 1). The 28‐day application time was the most common, accounting for more than 50% of the datasets. Within group variance values (*var*) were similar across different application times, with no clear pattern related to shorter or longer exposures. Overall, there was no evidence that application time impacts *var* for the DupSeq datasets (Figure 1D).

The DupSeq dose–response data included eight tissue types: liver (16 datasets), bone marrow (11), blood (2), lung (6), seminiferous tubules (8), kidney (2), and stomach and spleen (1 each) (Table 1). Liver and bone marrow were the most frequently represented, together accounting for more than 50% of the datasets. Within‐group variance values (*var*) across tissues fell within a similar range, with no observable tissue effect (Figure 1E).

The DupSeq datasets spanned various tissue sampling times, including 1 day (8 datasets), 2 days (2), 3 days (15), 24 days (3), 28 days (16), 42 days (1), and 70 days (2) (Table 1). The most common sampling times were 3 and 28 days, together making up more than 65% of the datasets. Within‐group variance values (*var*) remained similar across the different sampling times, with no clear trend in the available data. Overall, sampling time did not have an observable effect on *var* (Figure 1F).

Finally, we evaluated the effect of the DNA fragmentation method on *var*. The DupSeq datasets included two approaches: sonication (15 datasets) and enzymatic fragmentation (32 datasets) (Table 1). Variance values for these methods overlapped, with no consistent differences observed. Overall, the fragmentation method did not observably affect *var* (Figure 1G).

In summary, the results obtained indicated that the investigated experimental factors do not influence *var* estimates for the DupSeq technology (Figure 1). Importantly, the effects of several other experimental factors were also investigated (e.g., sex, animal age, and dose group size). Similar to what has already been noted, none of these variables influenced *var* estimates (data not shown). As noted, the data available for the other technologies (i.e., PECC‐Seq and Hawk‐Seq) are markedly insufficient for meaningful analysis regarding the influence of experimental factors on *var* (Tables S2 and S3).

**FIGURE 2:**
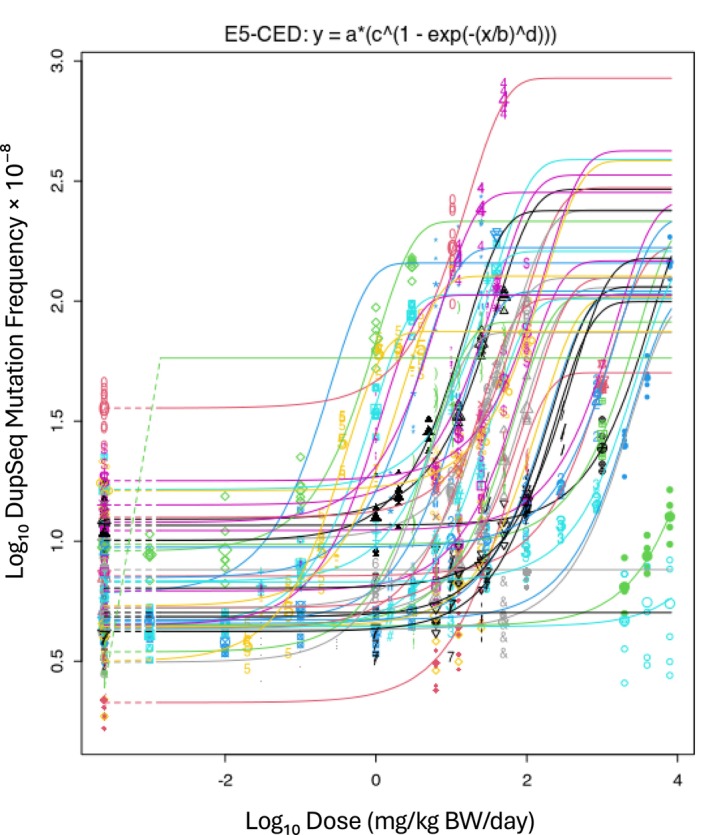
BMD analysis of the DupSeq data (47 datasets). Single *var* and *c* values were estimated across datasets, whereas parameters *a* and *b* were set as dataset‐dependent. The estimated *var* value (0.0783) is considered representative of the typical variance used for calculating the technology‐specific BMR (Equation 2).

Since the results illustrated in Figure 1 do not show evidence that the examined experimental factors affect *var*, the next step was to estimate a typical *var* value. Given the similarities between the various technologies examined, a single *var* value can also be assumed for Hawk‐Seq and PECC‐Seq. To achieve this, all available dose–response data for each specific ECS technology were analyzed together, allowing a single *var* estimate to be derived and used for calculating technology‐specific BMR values. These technology‐specific *var* estimates formed the basis for BMR calculation using the ES theory (Equation 2). For DupSeq, the 47 datasets yielded a *var* of 0.078 (90% CI: 0.073–0.084) (Figure 2). Figure S1 illustrates the dose–response curves for each of the analyzed datasets.

**FIGURE 3:**
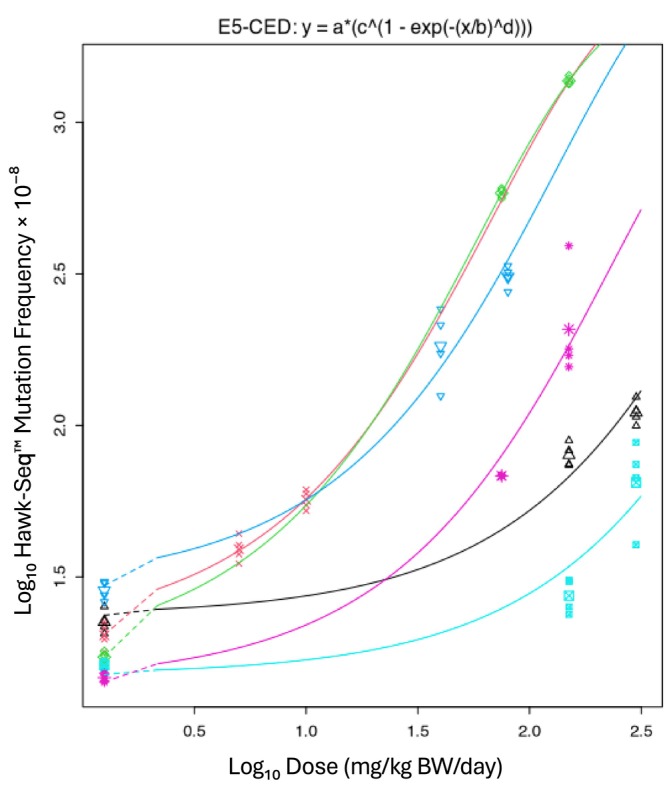
BMD analysis of the Hawk‐Seq data (6 datasets). Single *var* and *c* values were estimated across datasets, whereas parameters *a* and *b* were set as dataset‐dependent. The estimated *var* value (0.031) represents the typical variance used for calculating the technology‐specific BMR (Equation 2).

**FIGURE 4:**
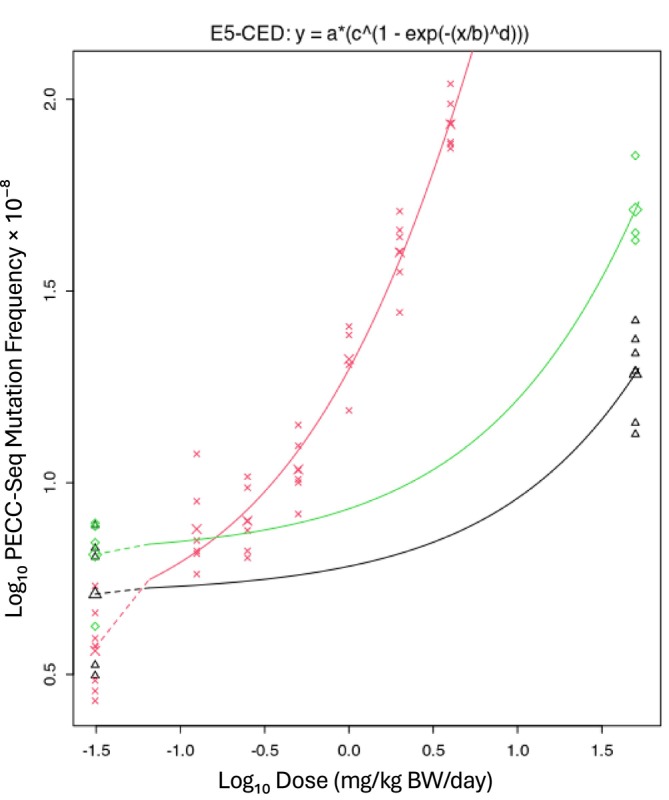
BMD analysis of the PECC‐Seq data (3 datasets). Single *var* and *c* values were estimated across datasets, whereas parameters *a* and *b* were set as dataset‐dependent. The estimated *var* value (0.057) represents the typical variance used for calculating the technology‐specific BMR (Equation 2).

The six Hawk‐Seq datasets yielded a *var* value of 0.031 (90% CI: 0.024–0.041), and the three PECC‐Seq datasets yielded a *var* value of 0.057 (90% CI: 0.043–0.079) (Figures 3 and 4). Figures S2 and S3 illustrate the dose–response relationships for each of the analyzed datasets.

Technology‐specific BMR values could, in principle, be derived from parameter *c*. However, scaling the BMR to *c* can be challenging in practice, as sometimes it can be difficult to obtain an estimate of *c* with acceptable precision. In contrast, *var* can generally be reliably estimated. For DupSeq, *c* was 17.2, with a relatively narrow CI of 15.9–18.6. In contrast, Hawk‐Seq had a *c* estimate of 150.3, with a broader CI of 90.7–436, and PECC‐Seq was highly uncertain, with a *c* of 3490 and an extremely wide CI of 40 to infinity. This variability in precision reflects the fact that the dose–response datasets for Hawk‐Seq and PECC‐Seq did not contain sufficient information to indicate where the dose–response relationships reach a plateau at higher doses. This causes PROAST to produce imprecise estimates of *c*. In contrast, *var* provides a more reliable basis for determination of technology‐specific BMR values. This is consistent with the approach recently employed by White et al. (2025).

Using the technology‐specific *var* values derived above, the ES theory was employed to calculate BMRs for each ECS technology. For DupSeq, analysis of 47 datasets resulted in a BMR of 27.7%. The six Hawk‐Seq datasets yielded a BMR of 16.6%, whereas the three PECC‐Seq datasets produced a BMR of 23.3% (Table 5). These values were obtained by applying Equation (2), using the square root of the estimated *var* (denoted as *s*) to reflect the typical within‐group variability for each technology (Figures 2, 3, 4). Unfortunately, HiFi data were not compatible with the ES approach. Although the published data included a study that examined three compounds, there was only a single shared control group. Consequently, as mentioned earlier, *var* values could not be determined, and HiFi results could only be used to calculate a BMR using the 1SD approach (see below).

**TABLE 5:** BMR values based on the Slob (2017) Effect Size (ES) approach.

ECS technology	*N* (datasets)	Var (90% CI)	BMR (%)
DupSeq	47	0.078 (0.073–0.084)	27.7
Hawk‐Seq	6	0.031 (0.024–0.041)	16.6
PECC‐Seq	3	0.057 (0.043–0.079)	23.3

We also calculated BMR values using the 1SD approach described by Zeller et al. (2017) (Table 6). This method uses study‐specific control standard deviations, truncated historical control standard deviations, or the median absolute deviation (MAD) values of historical controls. For DupSeq, the control data from 50 studies and 216 observations yielded BMRs of 22.6% (study‐specific SDs), 28.8% (historical control SD), and 24.3% (historical control MAD). Hawk‐Seq results, which were based on six studies and 22 observations, yielded BMR values of 5.6% (study‐specific SDs), 6.5% (historical control SD), and 13.8% (historical control MAD). For PECC‐Seq, three studies and 16 observations yielded BMR values of 29.3% (study‐specific SDs), 31.5% (historical control SD), and 28.7% (historical control MAD). Based on 11 observations from a single study, HiFi BMR values were 9.5% (study‐specific SD), 22.8% (historical control SD), and 17.2% (historical control MAD). These analyses show that BMR values for the four ECS technologies are well below the 50% value that is currently recommended by White et al. (2025) for the other in vivo mutagenicity endpoints.

**TABLE 6:** BMR values based on the Zeller et al. (2017) approach.

ECS technology	Study‐specific SD		Historical control SD^a^		MAD of historical controls^a^	
	*N* (studies)	BMR (%)	*N* (obs)	BMR (%)	N (obs)	BMR (%)
DupSeq	50	22.6	216	28.8	216	24.3
Hawk‐Seq	6	5.6	22	6.5	22	13.8
PECC‐Seq	3	29.3	16	31.5	16	28.7
HiFi	4	9.5	11	22.8	11	17.2

^a^ Based on the analysis of right‐truncated historical control distributions.

## 4 Discussion

Existing regulatory guidance provides only general recommendations for BMR selection, and there are no current recommendations for ECS technologies. To address this gap, we analyzed an in vivo ECS dose–response database using two approaches: the Slob (2017) ES theory and the 1SD method described by Zeller et al. (2017). Across DupSeq datasets, both approaches produced similar BMR estimates, supporting a consensus value of 30%. In contrast, BMRs for the other ECS technologies were more variable, ranging from 5.6% to 31.5% depending on the method and dataset availability. The paucity of available data prohibits any robust statements for the PECC‐Seq, Hawk‐Seq, and HiFi technologies.

The ES theory and 1SD approaches differ conceptually in how they define BMRs. The ES framework (Slob 2017) pools variance across studies to generate stable technology‐specific values, whereas the 1SD method (Zeller et al. 2017) relies on values reflecting variation in negative controls. The latter can be calculated more readily, but it is more sensitive to experimental noise. When applied to the ECS data, the two methods produced consistent estimates for DupSeq and PECC‐Seq. In contrast, greater divergence was observed for Hawk‐Seq and HiFi, where limited datasets made the 1SD approach more sensitive to variability in control values. These patterns reveal that the ES framework provides a more robust foundation for deriving ECS‐specific BMRs. However, additional data could improve the reliability of BMR estimates based on (historical) control data. The 1SD approach can serve as a useful complementary tool, particularly when decisive *var* values are difficult to determine due to data limitations (e.g., number of tested doses).

Earlier work that discussed in vivo mutagenicity endpoints, particularly the TGR and *Pig‐a* assays, supports higher BMR values, that is, rather than the aforementioned default 5% for continuous endpoints. The results presented in Slob (2017) yields BMR estimates in the 46%–74% range for these endpoints. More recently, extensive analyses of TGR and *Pig‐a* dose–response data by White et al. (2025) provided values in the 47%–60% range; the authors recommended a uniform BMR of 50% for in vivo mutagenicity endpoints. Interestingly, this value has already been used for interpretation of ECS dose–response data. Indeed, although this choice was not based on technology‐specific analyses such as those presented here, several recent works employed a BMR of 50% for their interpretation of ECS dose–response data (i.e., Lynch et al. 2024; Powley et al. 2024; Bercu et al. 2023; Zhang et al. 2024, 2025; Roper et al. 2025; Simon et al. 2025; Schuster et al. 2024).

Our new analysis of ECS technologies suggests that the default value of 50% may be too high. Across DupSeq, Hawk‐Seq, PECC‐Seq, and HiFi, the derived BMRs were consistently lower, reflecting the distinctive features of these platforms, for example, their ability to detect extremely low‐frequency mutations and their low and well‐controlled background variations. This finding may not come across as very surprising since both the ES theory and 1 SD approach take natural variability into account when deriving endpoint‐specific BMRs. However, it is important to note that the within‐group variation (*var*) in the data also unavoidably depends on experiment error. When compared to mutagenicity assessment technologies such as the TGR and *Pig‐a* assays (White et al. 2025), DupSeq, Hawk‐Seq, PECC‐Seq, and HiFi have substantially higher mutation frequency precision. This is likely attributable to the fact that, for example, DupSeq MFmin values are clonally corrected mutation frequency minimums. Indeed, Beal et al. (2015) noted increased precision of *lacZ* (MutaMouse) transgene mutations when the transgene was sequenced, and the mutant frequency values corrected for clonal expansion. Additionally, it is important to emphasize that, relative to the older TGR assays, the advanced ECS technologies are less prone to stochastic biological variability. Lastly, TGR assays are also prone to variability associated with the multistep transgene recovery, packaging and scoring process.

The substantially reduced measurement errors associated with ECS endpoints lead to reduced variability in the dose–response data, and the concomitantly lower BMRs shown herein. These findings indicate that direct application of a BMR of 50% does not adequately represent ECS technologies. Instead, the present work supports a more conservative BMR in the range of 30% for ECS technologies. This recommendation is consistent across technologies within the available data, supported by both the ES and 1SD approaches. Moreover, it remains within the broader range of BMR values reported for genotoxicity endpoints more generally (18%–79%; Slob 2017; Wills et al. 2017; Zeller et al. 2017; White et al. 2020). Importantly, as ECS datasets continue to become more available, these estimates can be refined and revisited to evaluate their alignment with BMR values used for other mutagenicity endpoints. This will provide further knowledge to determine whether deviation from the 50% default remains appropriate for regulatory interpretations of ECS dose–response data.

Importantly, the Slob ES theory indicates that, to be meaningful, critical effect size values (i.e., BMRs) should be scaled to the maximum effect size observed for a given endpoint. Furthermore, for risk assessment purposes, the effect size deemed to be *critical* (i.e., the critical effect size or CES) can be defined as a suitably small effect, where small is defined as 1/8 of the log‐transformed maximum effect for the endpoint. Alternatively, in cases where it is difficult to effectively determine the endpoint‐specific maximum effect size (i.e., *c*), the within group variance (i.e., *var*) can be employed according to Equation (2). Specifically, with respect to Equation (2), the effect size deemed to be critical is aligned with that indicated by Slob (2017), that is, 1/8 log‐transformed maximum. Unfortunately, it is not currently possible to objectively evaluate small effects in an adversity context; however, the same limitation applied to the BMR values based on analyses of *Pig‐a* and TGR dose–response data (White et al. 2025). That said, it is possible to compare the BMR estimates between the ECS endpoints investigated herein (Tables 5 and 6), and other in vivo mutagenicity assessment endpoints (i.e., 47%–60% for *Pig‐a* and TGR). With respect to the 1SD approach described by Zeller et al. (2017), it should be emphasized that the calculated BMR values reflect the minimum dose required to elicit a meaningful increase in MF above the negative control.

A key limitation of the current study is the uneven availability of ECS data across technologies. The DupSeq analyses were supported by 47 datasets, whereas Hawk‐Seq and PECC‐Seq were limited to six and three datasets, respectively. HiFi data were limited to a single study, and the available data could only be analyzed using the 1SD approach. This imbalance restricted the ability to determine technology‐specific BMR values for Hawk‐Seq, PECC‐Seq, and HiFi. There were no in vivo mutagenicity dose response data for new technologies such as SMM‐Seq (Maslov et al. 2022) and CODEC (Bae et al. 2023). As additional studies become available, particularly for Hawk‐Seq, PECC‐Seq, and HiFi, the reliability of BMR estimates can be improved. Until such time, the BMR values determined for Hawk‐Seq, PECC‐Seq, and HiFi technologies should be regarded as preliminary.

In summary, this study addresses the need for empirically determined BMR values for in vivo ECS mutagenicity assessment technologies. Based on technology‐specific estimates derived using both the ES theory and the 1SD approach, a default BMR of 30% is recommended. This value reflects a balanced, data‐driven choice that aligns with DupSeq‐derived estimates, and falls within the broader range of values observed across ECS technologies. Nevertheless, with respect to the other evaluated technologies (i.e., Hawk‐Seq, PECC‐Seq, and HiFi), the paucity of dose–response data indicates that the recommended BMR value should be regarded as provisional. Although it could be argued that a BMR value of 30% provides a consistent basis for quantitative interpretation of ECS dose–response data in a risk assessment context, it should be noted that risk assessments based on a BMR of 30% may lead to conservative conclusions relative to those aligned with a BMR of 50% (White et al. 2025). Nevertheless, it may be more pragmatic to use the recently recommended value of 50% since the difference in BMR might be regarded as inconsequential given the need to employ uncertainty factors (UFs) when calculating Health‐based Guidance Values (HBGVs) such as the PDE (Johnson et al. 2021; Simon et al. 2025). Similarly, the difference in BMR will have minimal impact on calculated MOE values (Chepelev et al. 2023). Overall, specification of an empirically derived BMR for ECS technologies will promote the integration of ECS data into quantitative dose–response assessments employed for human health risk assessment and regulatory decision‐making.

## Author Contributions

**Saron Mulugeta:** data collection, curation, analysis and interpretation, draft manuscript preparation. **Francesco Marchetti:** project conceptualization and supervision, data analysis and interpretation, manuscript reviewing and editing. **Guangchao Chen:** data analysis and interpretation, manuscript reviewing and editing. **Connie Chen:** project supervision, manuscript reviewing and editing. **Raechel Puglisi:** project supervision, manuscript reviewing and editing. **Paul A. White:** project conceptualization and supervision, data analysis and interpretation, manuscript reviewing and editing.

## Funding

This work was supported by Health Canada’s Chemicals Management Plan, the Government of Canada’s Genomics Research Development Initiative, and the Burroughs Wellcome Fund (1021737).

## Conflicts of Interest

The authors declare no conflicts of interest.

## Supporting information


**Figure S1:** PROAST output of the individual dose–response relationships for the 47 DupSeq datasets.
**Figure S2:** PROAST output of the individual dose–response relationships for the 6 Hawk‐Seq datasets.
**Figure S3:** PROAST output of the individual dose–response relationships for the 3 PECC‐Seq datasets.

**Tables S1–S3:** Summary of the dose–response data used for the presented analyses.

## Data Availability

The data that supports the findings of this study are available in the Supporting Information of this article.
