# Supplementary material for: Benchmark Response Values for Error‐Corrected Sequencing Mutagenicity Assessment Technologies
Source: Environ Mol Mutagen. 2026 Apr 27;67(4):e70051. doi: 10.1002/em.70051 (PMC13112513; doi:10.1002/em.70051)
Supplement: Supplementary file 1 — Figure S1: PROAST output of the individual dose–response relationships for the 47 DupSeq datasets. Figure S2: PROAST output of the individual dose–response relationships for the 6 Hawk‐Seq datasets. Figure S3: PROAST output of the individual dose–response relationships for the 3 PECC‐Seq datasets. [file EM-67-0-s001.pptx]

## Slide 1
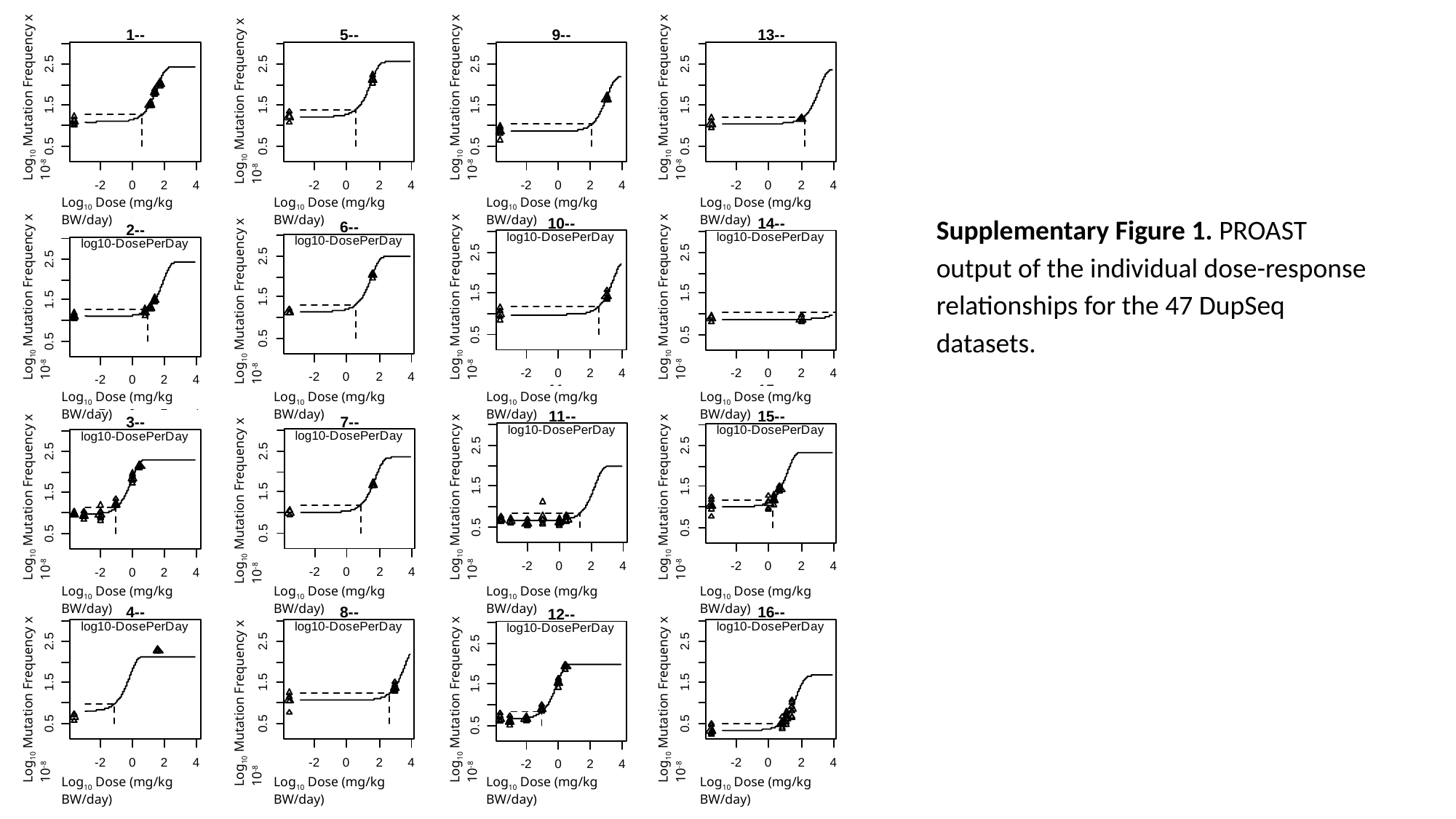

Log10 Mutation Frequency x 10-8
Log10 Mutation Frequency x 10-8
Log10 Mutation Frequency x 10-8
Log10 Mutation Frequency x 10-8
Log10 Dose (mg/kg BW/day)
Log10 Dose (mg/kg BW/day)
Log10 Dose (mg/kg BW/day)
Log10 Dose (mg/kg BW/day)
Log10 Mutation Frequency x 10-8
Log10 Mutation Frequency x 10-8
Log10 Mutation Frequency x 10-8
Log10 Mutation Frequency x 10-8
Log10 Dose (mg/kg BW/day)
Log10 Dose (mg/kg BW/day)
Log10 Dose (mg/kg BW/day)
Log10 Dose (mg/kg BW/day)
Log10 Mutation Frequency x 10-8
Log10 Mutation Frequency x 10-8
Log10 Mutation Frequency x 10-8
Log10 Mutation Frequency x 10-8
Log10 Dose (mg/kg BW/day)
Log10 Dose (mg/kg BW/day)
Log10 Dose (mg/kg BW/day)
Log10 Dose (mg/kg BW/day)
Log10 Mutation Frequency x 10-8
Log10 Mutation Frequency x 10-8
Log10 Mutation Frequency x 10-8
Log10 Mutation Frequency x 10-8
Log10 Dose (mg/kg BW/day)
Log10 Dose (mg/kg BW/day)
Log10 Dose (mg/kg BW/day)
Log10 Dose (mg/kg BW/day)
Supplementary Figure 1. PROAST output of the individual dose-response relationships for the 47 DupSeq datasets.

## Slide 2
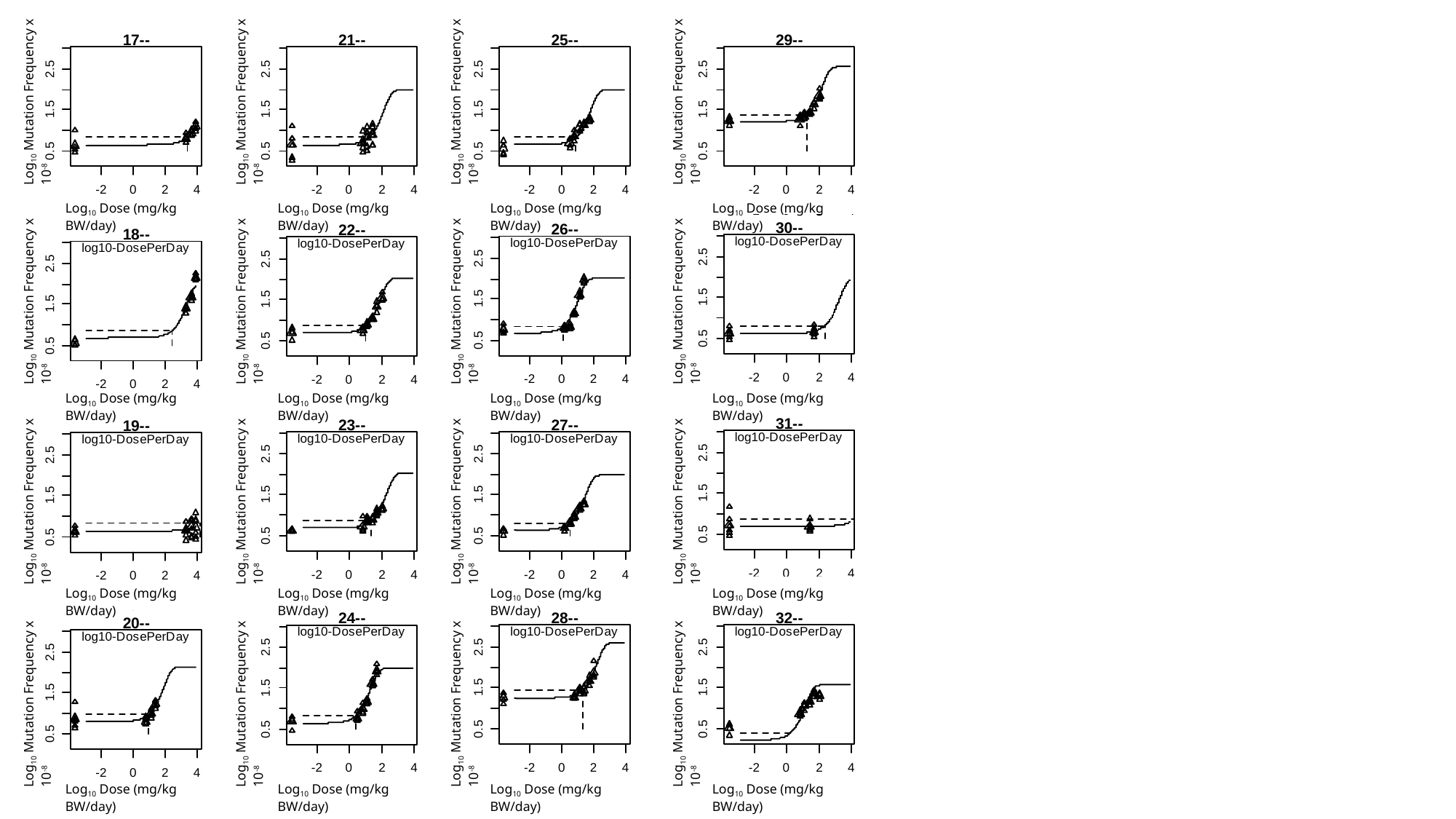

Log10 Mutation Frequency x 10-8
Log10 Mutation Frequency x 10-8
Log10 Mutation Frequency x 10-8
Log10 Mutation Frequency x 10-8
Log10 Dose (mg/kg BW/day)
Log10 Dose (mg/kg BW/day)
Log10 Dose (mg/kg BW/day)
Log10 Dose (mg/kg BW/day)
Log10 Mutation Frequency x 10-8
Log10 Mutation Frequency x 10-8
Log10 Mutation Frequency x 10-8
Log10 Mutation Frequency x 10-8
Log10 Dose (mg/kg BW/day)
Log10 Dose (mg/kg BW/day)
Log10 Dose (mg/kg BW/day)
Log10 Dose (mg/kg BW/day)
Log10 Mutation Frequency x 10-8
Log10 Mutation Frequency x 10-8
Log10 Mutation Frequency x 10-8
Log10 Mutation Frequency x 10-8
Log10 Dose (mg/kg BW/day)
Log10 Dose (mg/kg BW/day)
Log10 Dose (mg/kg BW/day)
Log10 Dose (mg/kg BW/day)
Log10 Mutation Frequency x 10-8
Log10 Mutation Frequency x 10-8
Log10 Mutation Frequency x 10-8
Log10 Mutation Frequency x 10-8
Log10 Dose (mg/kg BW/day)
Log10 Dose (mg/kg BW/day)
Log10 Dose (mg/kg BW/day)
Log10 Dose (mg/kg BW/day)

## Slide 3
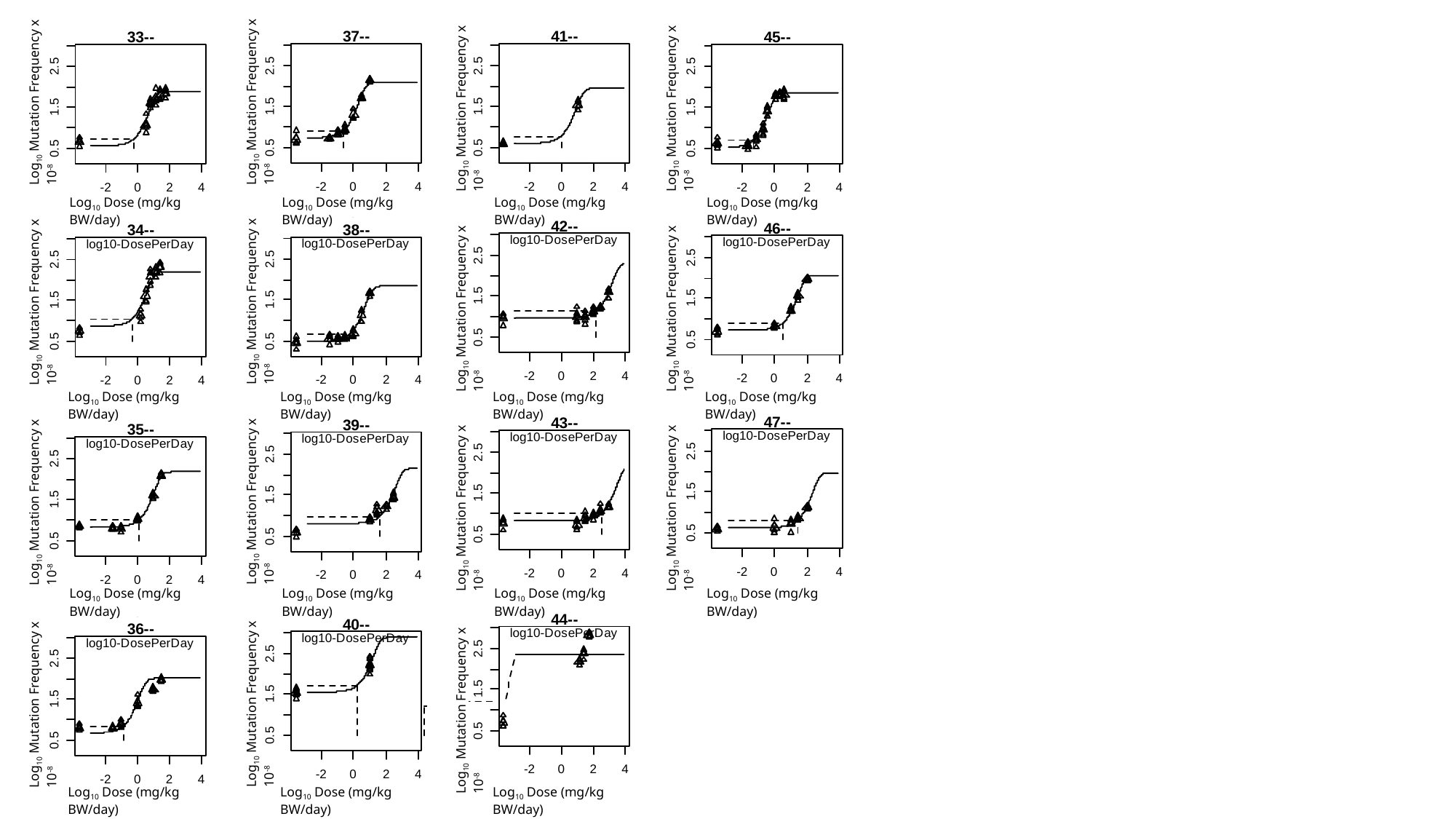

Log10 Mutation Frequency x 10-8
Log10 Mutation Frequency x 10-8
Log10 Mutation Frequency x 10-8
Log10 Mutation Frequency x 10-8
Log10 Dose (mg/kg BW/day)
Log10 Dose (mg/kg BW/day)
Log10 Dose (mg/kg BW/day)
Log10 Dose (mg/kg BW/day)
Log10 Mutation Frequency x 10-8
Log10 Mutation Frequency x 10-8
Log10 Mutation Frequency x 10-8
Log10 Mutation Frequency x 10-8
Log10 Dose (mg/kg BW/day)
Log10 Dose (mg/kg BW/day)
Log10 Dose (mg/kg BW/day)
Log10 Dose (mg/kg BW/day)
Log10 Mutation Frequency x 10-8
Log10 Mutation Frequency x 10-8
Log10 Mutation Frequency x 10-8
Log10 Mutation Frequency x 10-8
Log10 Dose (mg/kg BW/day)
Log10 Dose (mg/kg BW/day)
Log10 Dose (mg/kg BW/day)
Log10 Dose (mg/kg BW/day)
Log10 Mutation Frequency x 10-8
Log10 Mutation Frequency x 10-8
Log10 Mutation Frequency x 10-8
Log10 Dose (mg/kg BW/day)
Log10 Dose (mg/kg BW/day)
Log10 Dose (mg/kg BW/day)

## Slide 4
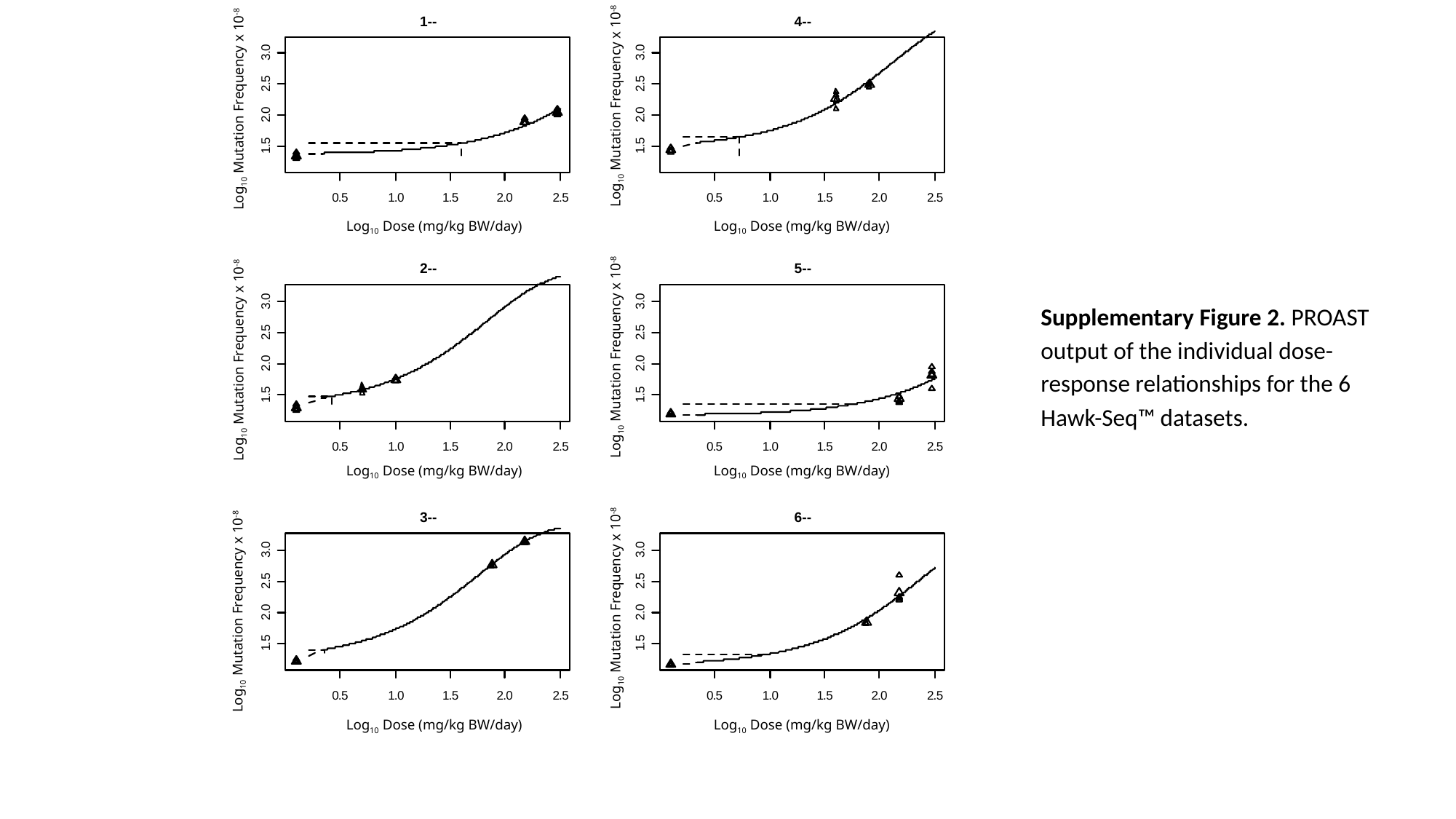

Log10 Mutation Frequency x 10-8
Log10 Mutation Frequency x 10-8
Log10 Mutation Frequency x 10-8
Log10 Mutation Frequency x 10-8
Log10 Mutation Frequency x 10-8
Log10 Mutation Frequency x 10-8
Log10 Dose (mg/kg BW/day)
Log10 Dose (mg/kg BW/day)
Log10 Dose (mg/kg BW/day)
Log10 Dose (mg/kg BW/day)
Log10 Dose (mg/kg BW/day)
Log10 Dose (mg/kg BW/day)
Supplementary Figure 2. PROAST output of the individual dose-response relationships for the 6 Hawk-Seq™ datasets.

## Slide 5
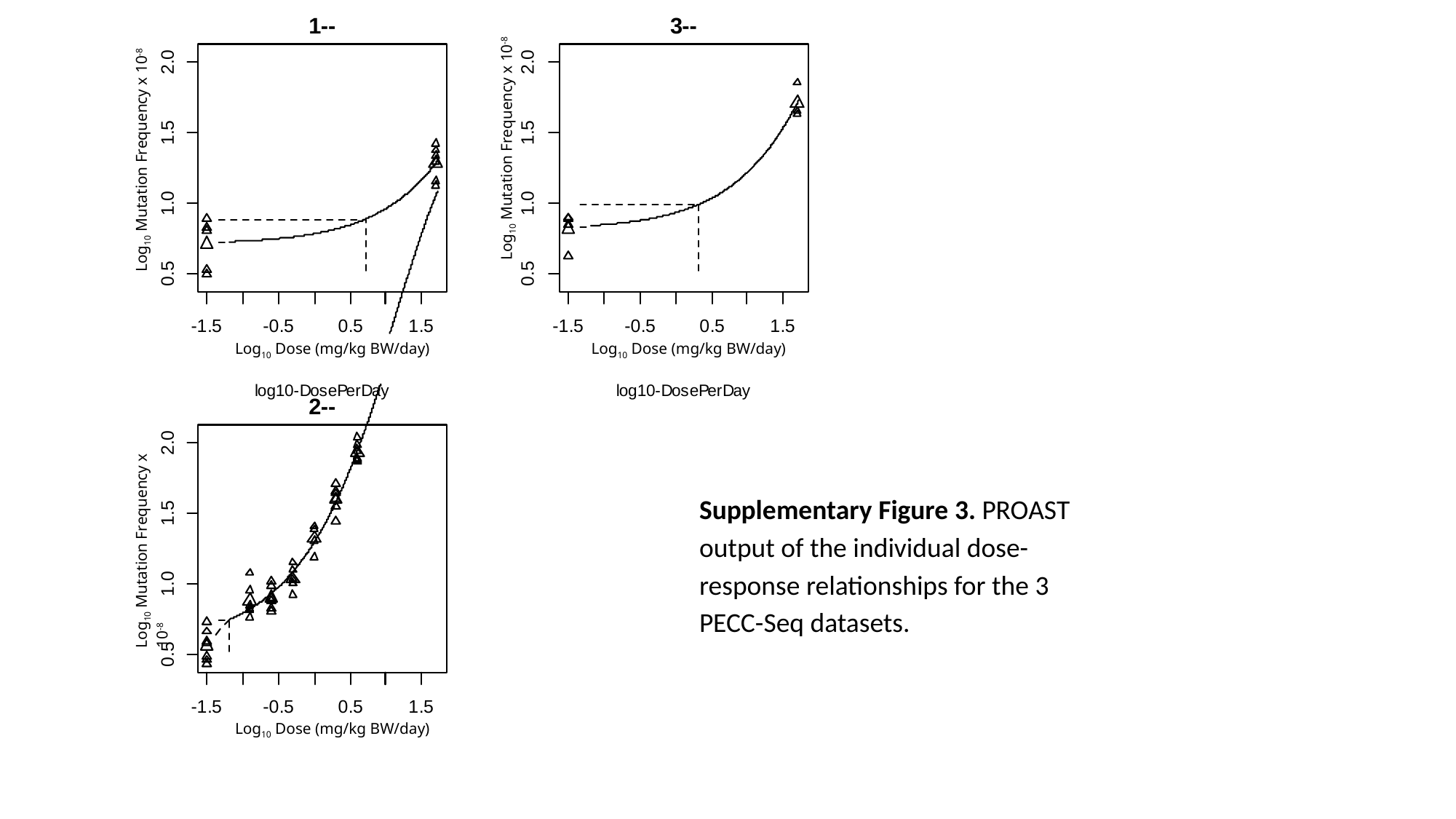

Log10 Mutation Frequency x 10-8
Log10 Mutation Frequency x 10-8
Log10 Dose (mg/kg BW/day)
Log10 Dose (mg/kg BW/day)
Log10 Mutation Frequency x 10-8
Log10 Dose (mg/kg BW/day)
Supplementary Figure 3. PROAST output of the individual dose-response relationships for the 3 PECC-Seq datasets.
